# Reduced Gray Matter Volume and Risk of Falls in Parkinson’s Disease with Dementia Patients: A Voxel-Based Morphometry Study

**DOI:** 10.3390/ijerph17155374

**Published:** 2020-07-26

**Authors:** Kai-Lun Cheng, Li-Han Lin, Po-Cheng Chen, Pi-Ling Chiang, Yueh-Sheng Chen, Hsiu-Ling Chen, Meng-Hsiang Chen, Kun-Hsien Chou, Shau-Hsuan Li, Cheng-Hsien Lu, Wei-Che Lin

**Affiliations:** 1 Department of Medical Imaging, Chung Shan Medical University Hospital, Taichung 402, Taiwan; chengkailun108@gmail.com; 2 School of Medical Imaging and Radiological Sciences, Chung Shan Medical University, Taichung 402, Taiwan; 3 Department of Veterinary Medicine, National Chung Hsing University, Taichung 402, Taiwan; 4 Department of Diagnostic Radiology, Kaohsiung Chang Gung Memorial Hospital, and Chang Gung University College of Medicine, Kaohsiung 833, Taiwan; cimetidine.tw@yahoo.com.tw (L.-H.L.); lovage@cgmh.org.tw (P.-L.C.); yssamchen@gmail.com (Y.-S.C.); suring.tw@gmail.com (H.-L.C.); sperfect1101@gmail.com (M.-H.C.); 5 Department of Physical Medicine and Rehabilitation, Kaohsiung Chang Gung Memorial Hospital, and Chang Gung University College of Medicine, Kaohsiung 833, Taiwan; ben0922852179@gmail.com; 6 Brain Research Center, National Yang-Ming University, Taipei 112, Taiwan; dargonchow@gmail.com; 7 Institute of Neuroscience, National Yang-Ming University, Taipei 112, Taiwan; 8 Department of Oncology and Hematology, Kaohsiung Chang Gung Memorial Hospital, and Chang Gung University College of Medicine, Kaohsiung 833, Taiwan; lee0624@adm.cgmh.org.tw; 9 Department of Neurology, Kaohsiung Chang Gung Memorial Hospital, and Chang Gung University College of Medicine, Kaohsiung 833, Taiwan; chlu99@cgmh.org.tw

**Keywords:** Parkinson’s disease, dementia, fall, brain structure, executive function

## Abstract

*Purpose*: Risk of falls is a common sequela affecting patients with Parkinson’s disease (PD). Although motor impairment and dementia are correlated with falls, associations of brain structure and cognition deficits with falls remain unclear. *Material and Methods*: Thirty-five PD patients with dementia (PDD), and 37 age- and sex-matched healthy subjects were recruited for this study. All participants received structural magnetic resonance imaging (MRI) scans, and disease severity and cognitive evaluations. Additionally, patient fall history was recorded. Regional structural differences between PDD with and without fall groups were performed using voxel-based morphometry processing. Stepwise logistic regression analysis was used to predict the fall risk in PDD patients. *Results:* The results revealed that 48% of PDD patients experienced falls. Significantly lower gray matter volume (GMV) in the left calcarine and right inferior frontal gyrus in PDD patients with fall compared to PDD patients without fall were noted. The PDD patients with fall exhibited worse UPDRS-II scores compared to PDD patients without fall and were negatively correlated with lower GMV in the left calcarine (*p*/*r* = 0.004/−0.492). Furthermore, lower GMV in the left calcarine and right inferior frontal gyrus correlated with poor attention and executive functional test scores. Multiple logistic regression analysis showed that the left calcarine was the only variable (*p* = 0.004, 95% CI = 0.00–0.00) negatively associated with the fall event. *Conclusions:* PDD patients exhibiting impaired motor function, lower GMV in the left calcarine and right inferior frontal gyrus, and notable cognitive deficits may have increased risk of falls.

## 1. Background

Common symptoms of Parkinson’s disease with dementia (PDD), including tremors, bradykinesia, rigidity, postural instability, memory impairment, and visual hallucinations can result in an increased risk of falls [1]. Postural instability, especially during gait initiation, is considered one of the primary factors leading to falls in patients with PDD. The instability worsens with visual impairment, as visual input contributes significantly to the maintenance of upright posture when walking. Other motor network disruptions, and cognitive decline may also affect patient balance, further increasing the risk of falls. Meanwhile, repeated falls can result in fractures requiring hospitalization, and in more serious cases, may be fatal, particularly in the elderly and female [2]. Furthermore, long-term complications include atrophy caused by disuse, lifestyle disruptions caused by fear of falls, or possible need for institutionalization [3]. To date, however, the relationship between disease severity or cognitive function and risks of falls in PDD patients has not yet to be further investigated.

In recent years, the application of neuroimaging in exploring the etiology of neurodegenerative diseases has gained in popularity, with Parkinson’s disease (PD) being one of the most commonly studied disorders. These imaging methods have contributed significantly to a broadening of our understanding of the disease, beyond simply impaired dopaminergic transmissions [4]. In fact, some neuroimaging studies have reported alterations in networks related to motor and cognitive symptoms [5,6,7,8,9,10]. Radiotracer imaging, such as positron emission tomography (PET) or single-photon emission computed tomography (SPECT), can be used to study the dopaminergic and other neurochemical systems [11]; while widely available magnetic resonance imaging (MRI) has demonstrated effectiveness at measuring anatomical changes of the brain, with several MRI studies reporting cortical atrophy in Parkinson’s patients with and without dementia [12,13,14,15], decreasing gray matter volume (GMV) of left calcarine in patients with impaired cognition in Huntington’s disease [16], or that of right inferior frontal gyrus in unaffected siblings of schizophrenia patients [17]. However, clarification of the pathophysiology within the brain and the association with risks of fall in PDD patients require further investigation.

In this study, we aim to relate the clinical disease severity and cognitive function of patients with PDD to the MRI findings. We hypothesized that PDD patients with histories of falls demonstrated relatively diminished physical and cognitive functions, which would be associated with distinct brain regions, different from those regions affected in PDD patients without histories of falls.

## 2. Materials and Methods

### 2.1. Subjects

Thirty-five patients with PDD (8 men and 27 women; mean age, 64.00 ± 5.85 years) and 37 age- and sex-matched healthy subjects (10 men and 27 women; mean age, 63.14 ± 5.34 years) were recruited for the study. Patients received MRI scans and neuropsychological tests (NPT) at the time of Parkinson disease diagnosis, while the control group received scans and tests at the initial enrollment in the study. Exclusion criteria for this study included a history of neurologic or psychiatric illness, the presence of developmental disorders, use of medication for unrelated conditions, and head injuries. The 35 PDD patients were further divided into two subgroups, based on the presence or absence of a history of falls (18 in the non-fall group, 3 men and 15 women; mean age, 62.78 ± 5.61 years; and 17 in the fall group, 5 men and 12 women; mean age, 65.29 ± 5.99 years). The Chang Gung Memorial Hospital Institutional Review Committees approved the study (IRB is 103-6906A3), and written informed consents were obtained from all subjects. 

### 2.2. Assessment of Clinical Disease Severity

The clinical features recorded were age at enrollment (or age at the time of the first reported symptom attributable to the disease), education level, and history of falls. Data on falls were prospectively collected from either the clinical records starting at enrollment or information provided by patients during the study period. The Morse fall scale assessed the risk of falling for hospital in-patients or those in long-term care and included considerations such as presence/absence of intravenous therapy [18]. Since all patients were enrolled in the study from the Neurology Out-patient Clinic, the scale was slightly modified by deletion of the item, “intravenous therapy or not”. The severity of PDD was graded according to the scores of the Unified Parkinson’s Disease Rating Scale (UPDRS), the Hoehn and Yahr staging, and activity of daily living assessment [19,20]. An experienced neurology nursing specialist who was blinded to the patients’ clinical and biochemical data was trained to measure these functional scores at the time of enrollment.

### 2.3. Neuropsychological Tests (NPT)

The NPT battery focusing on attention, executive function, speech and language, memory, and visuospatial functions were performed by a clinical psychologist blinded to each patient’s status. Attention was evaluated by letter number sequencing and digit span score from the Wechsler Adult Intelligence Scale-III (WAIS-III) [21], and by orientation score and attention score from the Cognitive Ability Screening Instrument (CASI) [22]. Executive functions were assessed using arithmetic, picture arrangement, digit symbol coding, and matrix reasoning scores from the WAIS-III, as well as abstract thinking scores from the CASI. Memory functions were assessed using short- and long-term memory scores from the CASI, and information scores from the WAIS-III. Speech and language ability were assessed using vocabulary, comprehension, and similarity scores from the WAIS-III, and language and semantic fluency scores from the CASI. Visuospatial functions were assessed using picture completion and block design scores from the WAIS-III and drawing score from the CASI. Raw scores for each NPT were transformed to *z* scores based on normative data. Cognitive domain scores were calculated by averaging *z* scores for NPT within specific domains, thereby accounting for any unequal distribution of tests per domain [23,24]. Impairment was defined as a *z* score ≤ 2 standard deviation (SD) compared to normal subjects for a given domain. PDD was defined as: (1) a diagnosis of idiopathic PD; (2) PD developed prior to the onset of dementia; (3) Mini-Mental State Examination (MMSE) < 26; (4) impairment in at least two of the neuro-psychological battery domains [25].

### 2.4. MRI Image Acquisition

All MRI scans were performed on the same 3-Tesla GE Signa whole-body MRI system (General Electric Healthcare, Milwaukee, WI, USA), equipped with an eight-channel head coil. A T1-weighted three-dimensional fluid-attenuated inversion-recovery fast spoiled gradient-recalled echo pulse sequence was used with the following imaging parameters for each participant and time-point: TR/TE/TI = 9.5/3.9/450 ms; flip angle, 15; NEX = 1; matrix size = 512 × 512; voxel size = 0.47 × 0.47 × 1.3 mm^3^; and 110 axial slices. An experienced neuroradiologist, blinded to the participants’ status, visually examined all the MRI scans to verify they were free from gross anatomical abnormalities. Subsequently, none of the participants in the study were excluded.

### 2.5. Voxel-Based Morphometry Processing

All structural MRI were post-processed by the same neuroradiologist, unaware of patients’ information. Voxel-based morphometry (VBM) was performed using Statistical Parametric Mapping software (SPM12) [1] running on Matlab 2015b (Matworks, Natick, MA, USA). The details of image processing and analysis of regional GMV differences were described in the Supplementary Materials (Method: Voxel-based morphometry processing and analysis).

### 2.6. Statistical Analysis

#### 2.6.1. Analysis of Demographic Data between Groups

Statistical analysis was performed using the statistics computer software SPSS 18 (SPSS Inc., Chicago, IL, USA). Descriptive statistics were expressed as mean ± SD for continuous variables, and as numbers for categorical variables. The clinical data of PDD patients and normal subjects were analyzed by chi-square test or independent *t* test where appropriate. One-way analysis of covariance (ANCOVA) was used to compare NPT with age, sex, and education as covariates. The threshold for statistical significance was *p* < 0.05. 

#### 2.6.2. Relationships among Disease Severity, Cognition Function, and Gray Matter Volume

For further relationship analysis, the regional GMV was extracted from the clusters with significant statistical difference from the PDD group comparisons. 

Partial correlation analysis, adjusted for age and sex, was performed to correlate GMVs showing significant differences in the PDD with fall and without fall groups with disease severity and NPT. Significance was set at a Bonferroni corrected *p* < 0.05, accounting for multiple region of interest (ROI) comparisons [26]. 

Stepwise logistic regression analysis with forward method was used to evaluate the relationships among disease severity, NPT, and areas with significant GMV differences in the PDD with fall and without fall groups, with adjustments made for other potential confounding factors.

## 3. Results

### 3.1. Clinical Characteristics, NPT, and Disease Severity among Groups

The demographic characteristics and NPT of PDD patients and normal controls are shown in Table 1. Except for age and sex, there were significant differences in education duration, MMSE, and NPT between the PDD patients and normal controls. All patients, regardless of fall status, performed significantly worse than those in the control group in all NPT (*p* < 0.05) (Table 1). There were no significant differences in MMSE and NPT between the PDD with fall group and the without fall group.

The disease severity of PDD patients with and without fall groups are shown in Table 2. Except for UPDRS-II (F = 2.540; *p* = 0.028), there were no significant differences in UPDRS, UPDRS I, UPDRS III, Hoehn and Yahr staging, activity of daily living assessment, and Morse fall scale between the PDD with fall and without fall groups.

### 3.2. Regional Gray Matter Volume (GMV) Aberrations among Groups

The regions with significant GMV differences are presented in Table 3.

#### 3.2.1. Comparison between all PDD Patients and Normal Controls

All PDD patients exhibited lower NPT results, and significantly lower GMV in the right superior temporal gyrus, left putamen, bilateral cerebellum, bilateral middle frontal gyri, right fusiform, bilateral precentral gyri, left superior frontal gyrus, left middle occipital gyrus, and left superior medial frontal gyrus (uncorrected *p* < 0.001) compared to the control group. 

#### 3.2.2. Comparison between PDD without Fall and Control Groups, and between PDD with Fall and Control Groups

By using a *p*-value of 0.001 (uncorrected), the PDD with fall group displayed lower GMV in the left middle temporal gyrus, left cuneus, left medial frontal gyrus, left middle occipital gyrus, bilateral inferior parietal gyri, right middle occipital gyrus, left middle temporal gyrus, right cingulate gyrus, and right anterior cingulate. Meanwhile, the PDD without fall group had lower GMV in the right cerebellum, and right superior temporal gyrus compared to the control group (uncorrected *p* < 0.001).

#### 3.2.3. Comparison between the PDD with Fall and without Fall Groups

Based on a nonstationary cluster extent threshold of *p* < 0.05 corrected for multiple comparisons with FWE, there was a significantly lower GMV in the left calcarine and right inferior frontal gyrus in PDD patient with fall group compared to PDD patients without fall group. 

**Figure 1:**
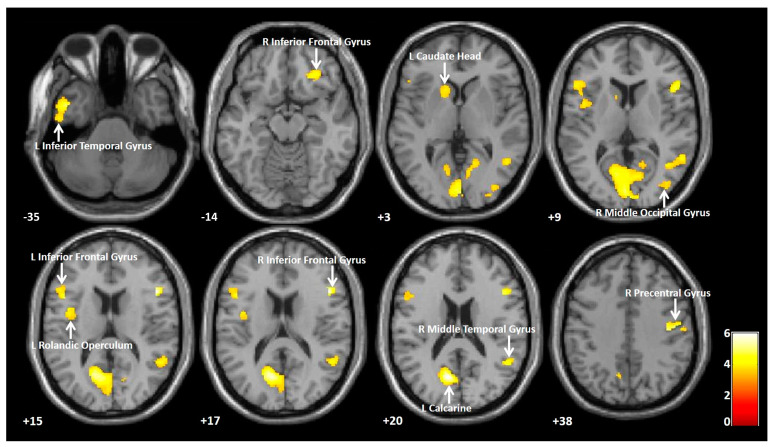
Lower gray matter volumes (GMVs) in PDD patients with fall vs without fall, with highlighted significant areas. Compared to PDD patients without fall, PDD patients with fall showed significantly lower GMV in the left inferior temporal gyrus, right inferior frontal gyrus, left caudate head, right middle occipital gyrus, left inferior frontal gyrus, left Rolandic operculum, right inferior frontal gyrus, right middle temporal gyrus, left calcarine, and right precentral gyrus. Among these areas, the clusters of left calcarine and R inferior frontal gyrus survived from statistical threshold of FWE-corrected *p* < 0.05.

By using an uncorrected *p*-value of 0.001, PDD with fall group showed significantly lower GMV in the left calcarine, right inferior frontal gyrus, left inferior temporal gyrus, right precentral gyrus, left inferior frontal gyrus, right middle temporal gyrus, right inferior frontal gyrus, left Rolandic operculum, left caudate head, and right middle occipital gyrus compared to PDD without fall group (Figure 1). 

### 3.3. Relationship among Disease Severity Profiles, NPT, and Extracted Regional GMV from PDD Patients 

**Figure 2:**
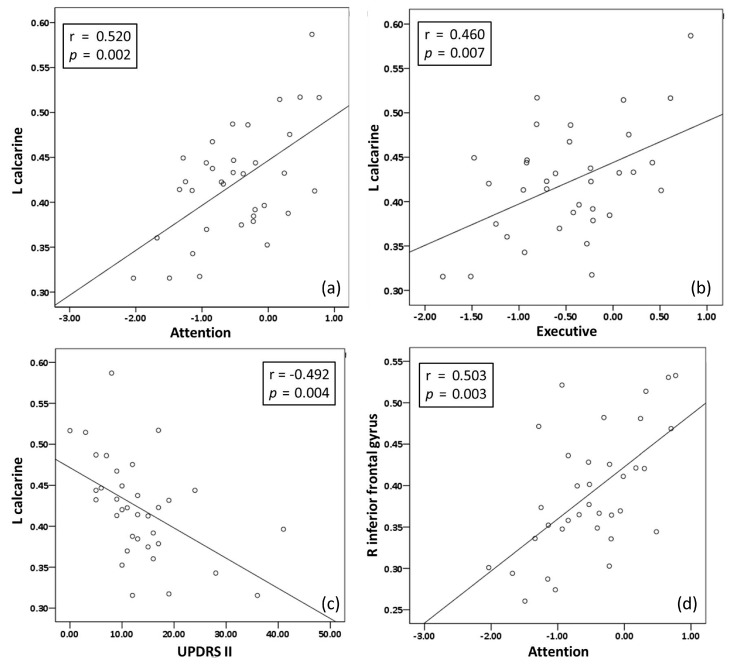
Correlations between NP tests (attention and executive) and UPDRS-II and regional GMV. (**a**) Left calcarine and attention. (**b**) Left calcarine and executive. (**c**) left calcarine and UPDRS-II. (**d**) Right inferior frontal gyrus and attention.

UPDRS-II was the only disease severity score showing significant difference between the PDD with fall and without fall groups; moreover, it exhibited a significantly negative correlation with a low GMV in the left calcarine (*p*/*r* = 0.004/−0.492) (Figure 2). 

There are positive correlations between NPT (attention and executive function) and the significantly lower GMV in PDD with fall group compared to PDD without fall group. The poor attention domain correlated with low GMV in the left calcarine (*p*/*r* = 0.002/0.520) and right inferior frontal gyrus (*p*/*r* = 0.003/0.503); while the low executive function correlated with low GMV in the left calcarine (*p*/*r* = 0.007/0.460) (Figure 2).

Multiple logistic regression analysis with forward method showed that the left calcarine was the only variable (*p* = 0.004, B: −37.6, 95% CI = 0.00−0.00) negatively associated with the fall event, meaning that lower GMV of the left calcarine could predict the occurrence of a fall event.

## 4. Discussion

### 4.1. Brief Summary and Potential Implications

There were significant differences in education level and the NPT between the PDD patients and controls, but no significant differences in the NPT between the PDD patients with and without fall groups. In the assessment of disease severity, the PDD with fall group only had significantly higher scores in UPDRS-II compared to the PDD without fall group. In the imaging study, all PDD patients showed significantly lower GMV in the right superior temporal gyrus, left putamen, bilateral cerebellum, bilateral middle frontal gyri, right fusiform, bilateral precentral gyri, left superior frontal gyrus, left middle occipital gyrus, and left superior medial frontal gyrus compared to the control group. There was a significantly lower GMV in left calcarine, and right inferior frontal gyrus in the PDD with fall group compared to the PDD without fall group. In terms of relationships, UPDRS-II showed a negative correlation with GMV in the left calcarine. Attention function was positively correlated with the GMV in the left calcarine, and right inferior frontal gyrus, while executive function was positively correlated with the GMV only in the left calcarine. Of note, the regression analysis showed that the GMV of the left calcarine was negatively associated with fall events.

Studies have reported the use of NPT to assess clinical characteristics of PDD patients [27,28,29]. Although differences existed in the evaluation tools, declining functions in many domains of NPT were noted in PDD patients, similar to the results reported herein. However, the specific domain accounting for falls has yet to be clarified. Although Papapetropoulos et al. [30] reported that PDD was one of the independent risk factors attributing to falls, no NPT were performed in the study. Another study found that mild cognitive impairment was related to increasing risk of fall, but the cognitive functions of the participants were rated by Clinical Dementia Rating Scale, which focused on the ability for self-care [31]. Meanwhile, Allcock et al. [32] reported on a relationship between impaired attention and fall frequency due to increasing difficulty in performing concurrent tasks and compensatory movements to prevent falls in PD patients. One systematic review also supported the concept that cognitive impairment, especially in the domains of attention and executive function, can disturb multitasking and thereby result in falls [33]. In this study, complete NP tests were assessed for all participants, and the PDD patients had significantly lower z scores compared to the controls. Although the z scores of most domains of the NPT in the PDD with fall group appeared to be lower than that of the PDD without fall group, no statistical difference was noted, possibly attributable to the small sample size.

In this study, multiple disease severity scales, including UPDRS, UPDRS I, II, III, Hoehn and Yahr stage, activity of daily living, and Morse fall scale, were assessed for all PDD participants. Although the disease severity in the PDD with fall group appeared to be severer than that of the PDD without fall group, no statistical difference was noted except for UPDRS-II, possibly attributable to the small sample size or similarity of disease severity of PDD participants. One systematic review reported that disease severity as measured by Hoehn and Yahr stage or by the UPDRS was found to be significantly associated with recurrent falls in PD [34]. Matinolli et al. [35] reported that UPDRS-II was a significant risk factor for predicting fall in PD, similar to the results reported herein. The UPDRS-II, specific for motor aspects of experiences of daily living, has 13 self-assessed items, including freezing when walking, a phenomenon closely related to fall. These results demonstrate UPDRS-II might be a tool to assess the fall risk in PD.

Many structural MRI studies have reported on the existence of cortical atrophy in PD patients, particularly atrophy of the hippocampus and the amygdala in patients with PD with or without dementia [12,13,14,15]. Recently, VBM brain MRI has been used in the study of patients with PD. These studies have demonstrated that patients with PDD present with limbic and widespread neocortical gray matter loss, while Parkinson’s patients without dementia mainly present with atrophy in the frontal and temporal regions [36,37,38,39]. Cristina et al. had found that PDD with visual hallucination presented gray matter volumetric reductions in the left orbitofrontal lobe when compared to normal subjects [2]. In the present study, the GMV of most neocortex regions of the PDD patients were less than those of the control group. The PDD with fall group mainly showed significantly decreased GMV in the left frontal, temporal, and occipital lobes, compared to the control group, similar to the results of other studies. Prior to this study, no imaging study had focused specifically on regions of the brain associated with fall risk. However, this study finds that the PDD with fall group had significantly less GMV in the left calcarine and right inferior frontal gyrus than that of the PDD without fall group. This finding is similar to a study comparing the brain structures of PD patients with and without rapid eye movement sleep behavior disorder [40]. Furthermore, the association between sleep disorders and falls in PD patients has been reported in a separate case-control study [41]. It is believed that sleep disorders can increase the risk of falls via several mechanisms, such as poor attention, slower reaction time, and impaired balance.

The calcarine region, part of the occipital lobe, is where the primary visual cortex is concentrated. The relationship between visuospatial ability and motor function has been demonstrated in previous studies [42,43]. Consistent with a previous study [44], this study demonstrates a negative correlation in PD patients between the GMV of the left calcarine and the UPDRS-II, which tests and rates the motor aspects associated with daily activities. Moreover, we identify associations between the GMV of the left calcarine and attention, implying a relationship between visual function and attention in PDD patients. Stuart et al. [45] conducted a cohort study and built a structural equation model showing associations between attention, visual function, saccade frequency, and gait impairment in PD patients. The abovementioned factors may result in poor executive function. Of note, the association between the GMV of the right inferior frontal gyrus and executive function is exhibited in this study, consistent with a previous imaging study which reported on the important role of the right inferior frontal gyrus in executive control [46]. Taken together, the results of the present study and separate studies suggest that visual function and specific domains of cognitive impairment play critical roles in fall events.

### 4.2. Limitations

Although this study demonstrates that specific domains of cognitive impairment and brain regions are reported to be related to falls in PDD patients, some limitations need to be addressed. First, this study applies a cross-sectional design, and further causal relationships should be investigated by future cohort study. Second, the small sample size limits the statistical power of demonstrating differences among groups. Third, the male to female odds ratio of developing dementia in PD patients between 65 and 69 years old has been reported to be 2:1 by a large cross-sectional study [47]; however, in this study, the number of female participants is higher than that of the male participants. This kind of selection bias may deviate our results from the real-world evidence, and future investigators should consider this when designing any future clinical trial.

## 5. Conclusions

Those PDD patients with falls may suffer from higher degrees of structural and functional degeneration of the brain, especially in the calcarine and inferior frontal regions. These brain regions play important roles in attention and visual function, and aberrations in these regions may be used to predict future risk of falls in PDD patients. The findings of this study suggest that closer attention to these comorbidities be paid in clinical practice for early intervention and prevention of fall events.

## Figures and Tables

**Table 1:** Demographic characteristics of PDD patients and normal controls.

Demographics	Normal Group	PDD			F +	*p*-Value +
		With Fall	Without Fall	All Patients		
Number of cases	37	17	18	35		
Sex (*n* = men/women)	10/27	5 / 12	3 / 15	8/27		0.630
Ages (years)	63.1 ± 5.34	65.3 ± 5.99	62.8 ± 5.61	64.0 ± 5.85	1.112	0.335
Education	9.95 ± 4.73 ^#^	5.53 ± 4.14 ^#^	6.72 ± 4.78	6.14 ± 4.45	6.416	0.003
MMSE	26.7 ± 2.22^§#^	19.2 ± 4.10 ^#^	21.1 ± 4.30 ^§^	20.1 ± 4.25	36.481	<0.001
**Neuropsychological domains**						
Attention	0.46 ± 0.48 ^§ #^	−0.70 ±-0.66 ^#^	−0.30 ± 0.72 ^§^	−0.49 ± 0.71	5.871	<0.001
Executive function	0.43 ± 0.76 ^§ #^	−0.54 ± 0.62 ^#^	−0.41 ± 0.65 ^§^	−0.48 ± 0.63	1.461	<0.001
Memory	0.41 ± 0.53 ^§ #^	−0.54 ± 0.85 ^#^	−0.34 ± 0.65 ^§^	−0.43 ± 0.75	2.140	0.001
Speech and language	0.46 ± 0.71 ^§ #^	−0.52 ± 0.58 ^#^	−0.50 ± 0.69 ^§^	−0.51 ± 0.63	0.317	<0.001
Visuospatial function	0.46 ± 0.62 ^§ #^	−0.45 ± 0.77 ^#^	−0.53 ± 0.74 ^§^	−0.49 ± 0.74	1.054	<0.001

F^+^ and *p* ^+^ represent the comparison between all PDD patients vs. the control group. Sex was compared by chi-square test. Age, education, and MMSE data were compared by independent *t* test. NP data were compared by ANCOVA after controlling for age, sex, and education. Data are presented as mean ± SD. ^#^ Significant differences between the control group and the PDD with fall group. ^§^ Significant differences between the control group and the PDD without fall group.

**Table 2:** Disease severity of PDD patients.

Disease Severity	PD with Dementia			F ^+^	*p*-Value ^+^
	With Fall	Without Fall	All Patients		
**Number of Cases**	17	18	35		
Unified Parkinson’s Disease Rating Scale (UPDRS) ^a^	57.0 ± 28.3	42.6 ± 22.9	49.6 ± 26.3	0.559	0.106
UPDRS I ^b^	4.88 ± 2.91	3.72 ± 2.95	4.28 ± 2.95	0.040	0.250
UPDRS-II ^c^	16.7 ± 9.98	10.5 ± 5.54	13.5 ± 8.49	2.540	0.028 *
UPDRS III ^d^	35.4 ± 16.8	28.3 ± 16.1	31.8 ± 16.6	0.010	0.213
Hoehn and Yahr staging	2.53 ± 0.89	2.14 ± 0.98	2.33 ± 0.95	0.294	0.228
Activity of daily living	76.5 ± 15.0	80.6 ± 16.3	78.6 ± 15.6	0.022	0.446
Morse fall scale	62.9 ± 27.7	49.7 ± 26.8	56.1 ± 27.7	0.102	0.161

F ^+^ and *p* ^+^ represent the comparison between PDD with fall group vs. PDD without fall group. ^a^ Total UPDRS score is the combined sum of parts I, II, and III. Theoretical minimum and maximum values are 0 and 176, respectively (176 represents the worst disability and 0 no disability).^b^ I. Mentation, behavior, and mood. Theoretical minimum and maximum values are 0 and 16, respectively. (16 represents the worst disability and 0 no disability). ^c^ II. Activities of daily living (ADL). Theoretical minimum and maximum values are 0 and 52, respectively. (52 represents the worst disability and 0 no disability). ^d^ III. Motor examination. Theoretical minimum and maximum values are 0 and 108, respectively. (108 represents the worst disability and 0 no disability). UPDRS, UPDRS I/II/III, Hoehn and Yahr staging, Activity of daily living, and Morse fall scale were compared by independent *t* test. Data are presented as mean ± SD.* Significant differences between the PDD with fall group and PDD without fall group.

**Table 3:** Regions of statistically significant lower GMV in PD with dementia patients compared to those of control.

Gray Matter Volume	Anatomical Regions	x	y	z	Cluster Size	T-Value
**Normal > all PDD**						
	R superior temporal gyrus *	59	−11	0	14770	5.90
	L putamen *	−27	15	6	10899	5.68
	R cerebellum *	20	−74	−47	14753	5.54
	R middle frontal gyrus *	29	50	8	591	5.40
	L middle frontal gyrus *	−29	42	30	304	4.94
	R fusiform	41	−57	−18	289	4.55
	R precentral gyrus	53	-8	47	711	4.51
	L precentral gyrus	−44	6	47	646	4.42
	L superior frontal gyrus	−18	60	8	445	4.26
	L middle occipital gyrus	−33	−81	14	195	4.20
	L superior medial frontal gyrus	−11	4	−20	238	4.11
	L cerebellum	−30	−68	-36	648	3.52
**Normal > PDD with fall**						
	L middle temporal gyrus *	−47	2	−35	53327	7.82
	L cuneus *	−5	−86	3	28263	7.14
	L medial frontal gyrus *	−12	39	18	2027	5.34
	L middle occipital gyrus	−33	−81	14	641	6.10
	R inferior parietal gyrus	57	−56	44	270	5.23
	L inferior parietal gyrus	−59	−47	45	281	4.79
	R middle occipital gyrus	42	−80	8	532	4.67
	L middle temporal gyrus	−41	−65	12	441	4.40
	R cingulate gyrus	14	11	44	165	3.92
	R anterior cingulate	14	44	3	234	3.87
**Normal > PDD without fall**						
	R cerebellum	17	−74	−45	1860	4.00
	R superior temporal gyrus	62	2	−3	236	3.63
**PDD with fall < PDD without fall**						
	L calcarine *	−9	−68	20	3735	5.97
	R inferior frontal gyrus *(pars triangularis)	48	20	17	333	6.01
	L inferior temporal gyrus	−47	3	−35	717	4.97
	R precentral gyrus	41	−15	38	363	4.77
	L inferior frontal gyrus (pars opercularis)	−47	15	15	444	4.73
	R middle temporal gyrus	50	-53	20	390	4.52
	R inferior frontal gyrus (pars orbitalis)	30	35	−14	385	4.32
	L Rolandic operculum	−42	2	15	312	4.06
	L caudate head	−14	15	3	248	3.82
	R middle occipital gyrus	41	−78	9	205	3.72

Voxel-based morphometry of PDD patients compared to those of controls at an uncorrected *p*-Value (<0.001). * Indicated for statistical threshold: uncorrected *p* < 0.001 with a cluster extent correction family-wise error (FWE)-corrected *p*-Value < 0.05. (x, y, and z) refer to the Montreal Neurological Institute coordinates. The T-value is determined by dividing the estimated regression coefficient by its standard error. Abbreviations: R Right; L Left.

## Supplementary Materials

The following are available online at https://www.mdpi.com/1660-4601/17/15/5374/s1, Method: Voxel-based morphometry processing and analysis.
