# Supplementary material for: Reduced Gray Matter Volume and Risk of Falls in Parkinson’s Disease with Dementia Patients: A Voxel-Based Morphometry Study
Source: Int J Environ Res Public Health. 2020 Jul 26;17(15):5374. doi: 10.3390/ijerph17155374 (PMC7432132; doi:10.3390/ijerph17155374)
Supplement: Supplementary file 1 [file ijerph-17-05374-s001.pdf]

Supplementary Materials and Methods

Voxel-Based Morphometry Processing

The 3D MRI datasets of all PDD patients and controls were processed using Statistical Parametric Mapping 12 (SPM12, http://www.fil.ion.ucl.ac.uk/spm) as detailed in the following steps. For better registration, all native space volumetric structural T1-weighted images were reoriented with the origin set close to the anterior commissure. Each participant's original image was then segmented into grey matter (GM), white matter (WM), and cerebrum spinal fluid (CSF) using the New Segment procedure in SPM12, a segmentation which provides an acceptable substitute for labor-intensive manual estimates [1], and the DARTEL inside SPM12 was used to spatially normalize the segmented images [2]. The image intensity of each voxel was modulated by the Jacobian determinants to ensure that regional differences in the total amount of GMV were conserved. To achieve normalization, the registered images were then transformed to Montreal Neurological Institute (MNI) space using affine spatial normalization. The normalized, modulated, and segmented GM images were then re-sliced by 1.5 mm × 1.5 mm × 1.5 mm voxels and smoothed using a Gaussian kernel of 8 mm full-width at half-maximum. For each individual, total GM, WM, and CSF were obtained and used to calculate the individual Total Intracranial Volume (TIV) by summing the volumes of the three mentioned components (GM, WM and CSF).

Analysis of Regional Gray Matter Volume Differences

Smoothed, modulated gray matter segments were analyzed with SPM12 within the framework of a General Linear Model (GLM). ANCOVA was performed with the covariations of age, sex, and TIV to investigate differences in regional GM volume between the two groups. All voxels with a GM probability value < 0.2 (range, 0–1) were eliminated to avoid possible partial volume effects around the margin between the GW and the WM. Nonstationary correction (part of the VBM toolbox), for correcting non-isotropic smoothness of the data, was used to investigate group differences [3]. The differences in GM volume were compared between the following groups: (1) all PDD patients vs. control group;(2) PDD without fall group vs. control group; (3) PDD with fall group vs. control group; and (4) PDD with fall group vs. PDD without fall group.

Due to the exploratory design of this study, strict criteria were used to obtain the findings. Of note, low voxel-level thresholds (uncorrected $p < 0.05$) might sensitize the cluster inference for spatially extended and lower spatial resolutions; by contrast, high (uncorrected $p < 0.001$) contiguous voxel thresholds with a cluster size >50 might generate a higher spatial cluster resolution but result in the loss of spatial extent. Therefore, the voxel level threshold for this study was set to an uncorrected $p < 0.001$, and a nonstationary cluster extent threshold of $p < 0.05$ corrected for multiple comparisons with family-wise error (FWE) [4] correction were used to obtain precise findings with higher spatial cluster resolutions. Anatomic structures of the coordinates representing significant clusters were identified based on the Talairach and Tournoux atlas [5].

References

1. Malone, I.; Leung, K.K.; Clegg, S.; Barnes, J.; Whitwell, J.L.; Ashburner, J.; Fox, N.; Ridgway, G.R. Accurate automatic estimation of total intracranial volume: A nuisance variable with less nuisance. *NeuroImage* **2015**, *104*, 366–372, doi:10.1016/j.neuroimage.2014.09.034.
2. Ashburner, J. A fast-diffeomorphic image registration algorithm. *NeuroImage* **2007**, *38*, 95–113, doi:10.1016/j.neuroimage.2007.07.007.
3. Hayasaka, S.; Phan, K.; Liberzon, I.; Worsley, K.J.; Nichols, T.E. Nonstationary cluster-size inference with random field and permutation methods. *NeuroImage* **2004**, *22*, 676–687, doi:10.1016/j.neuroimage.2004.01.041.
4. Lin, W.-C.; Chou, K.-H.; Chen, H.-L.; Huang, C.-C.; Lu, C.-H.; Li, S.-H.; Wang, Y.-L.; Cheng, Y.-F.; Lin, C.-P.; Chen, C.-C. Structural deficits in the emotion circuit and cerebellum are associated with depression, anxiety and cognitive dysfunction in methadone maintenance patients: A voxel-based

morphometric study. *Psychiatry Res. Neuroimaging* **2012**, *201*, 89–97, doi:10.1016/j.pscychresns.2011.05.009.
5. Talairach, J.; Tournoux, P. Co-planar stereotaxic atlas of the human brain. 3-Dimensional proportional system: an approach to cerebral imaging (1988).
